# Nonhuman treatment reduces helping others: self-dehumanization as a mechanism

**DOI:** 10.3389/fpsyg.2024.1352991

**Published:** 2024-03-05

**Authors:** Zaixuan Zhang, Zhansheng Chen

**Affiliations:** Department of Psychology, The University of Hong Kong, Pokfulam, Hong Kong SAR, China

**Keywords:** prosociality, self-dehumanization, dehumanization, objectification, interpersonal relation

## Abstract

Objectification is a daily experience with various negative consequences. In four studies (*N* = 877), we tested whether and how objectification experience contributes to decreased prosociality. Using correlational designs (Studies 1 and 2), we found that participants’ objectification experience negatively predicted their prosocial intention and that self-dehumanization could account for the negative association between objectification and prosocial intention. Next, by manipulating participants’ objectification experience, we found the negative effect of objectification on prosocial intention, as well as the mediating role of self-dehumanization (Studies 3 and 4). Additionally, we tested the mediating role of self-dehumanization in comparison with relative deprivation (another potential mediator), and consistently found that self-dehumanization was a stronger mediator in accounting for the effect of objectification on prosocial intention (Studies 1, 2, and 4). Together, our findings support the process of self-dehumanization following objectification and offer new insights into the relationship between objectification and prosociality. The implications and limitations of the research were discussed.

## Introduction

Objectification happens when people are treated as mere objects (Nussbaum, 1995), which results in a range of negative outcomes. For instance, punishments are more likely to be imposed on people suffering from objectification (Landau et al., 2012). Additionally, people are more willing to behave immorally toward those objectified targets (Wang and Krumhuber, 2017). More recently, Belmi and Schroeder (2021) found that being objectified during work reduces employees’ job satisfaction. However, relatively less convincing internal processes have been proposed or detected to interpret those harmful effects of objectification.

Previously, Fredrickson and Roberts (1997) theorized that objectification could be internalized by individuals and result in self-objectification. Empirically, Andrighetto et al. (2018) found that employees who are objectified during work tend to employ more self-objectification, and that such a pattern could then increase their conforming behaviors. It suggested that the internalization of objectification could help us understand some consequences of objectification. Because dehumanization is one significant component of objectification (Baldissarri et al., 2022), it could also be internalized by objectified victims. In the current research, we aimed to examine whether individuals will experience self-dehumanization after being objectified. Moreover, we aimed to test whether such self-dehumanization following objectification could reduce prosociality. By doing so, we hope to offer a novel perspective to understand those negative effects of objectification.

### Objectification and self-dehumanization

When people are objectified, they are denied of personhood and are treated merely as things or tools that can aid others’ goal achievement, and their emotions, needs, and feelings are all neglected. Previously, most of the scholars focused on the sexual aspect of objectification, especially sexual objectification toward females. For example, Fredrickson et al. (1998) argued that cultural practices of sexual objectification can heighten females’ preoccupation with their appearance, giving rise to feelings of body shame and anxiety. In recent years, nonsexual objectification has garnered significant attention. For example, Cheng et al. (2022) found that objectification experience could make individuals feel less authentic and that such limited authenticity could further decrease their subjective well-being. Poon et al. (2020) found that objectification in various social contexts could consistently elevate interpersonal aggression. Additionally, Zhang et al., (2023) showed that objectification can diminish individuals’ prosociality, which results from the sense of relative deprivation induced by objectification.

Objectification could also shape individuals’ self-perceptions. According to the theory of Looking-Glass Self (Cooley, 1902), people’s self-perception is partially reliant on how others see and treat them. Thus, victims of objectification and dehumanization may experience self-objectification and self-dehumanization. Fredrickson and Roberts (1997) argued that females might internalize the social culture of objectification and employ more self-objectification. Studies on sexual objectification have shown the impacts of the sexualization environment on individuals’ self-objectification (e.g., Karsay et al., 2018). Recently, researchers further found that nonsexual objectification experience could also lead to self-objectification (Baldissarri et al., 2021; Sainz and Baldissarri, 2021).

Self-objectification and self-dehumanization could be attributed to the complex nature of objectification. Baldissarri et al. (2022) theorized that dehumanization is a significant component of objectification, together with instrumentality. Specifically, individuals subjected to objectification are not only regarded as tools to serve the interests of those objectifying them, but their autonomy and subjectivity are also disregarded, relegating them to the status of nonhuman beings devoid of agency and emotions. That is, during an objectification experience, individuals suffer from instrumentalization and dehumanization simultaneously, which makes it different from a dehumanization experience, in which individuals’ humanness is denied, and their instrumentality is not the focus or relevant. Previously, Nussbaum (1995) argued that objectification involves the denial of autonomy and subjectivity, where objectified individuals are viewed as lacking the capacity to think, make decisions for themselves, and have their feelings and emotions disregarded. This implies that objectified individuals are treated as objects devoid of the ability to exercise cognitive autonomy and decision-making, whereas their emotions and feelings are similarly dismissed. As demonstrated by Haslam (2006), the capabilities to feel with emotions and act with agency are both crucial traits that make an individual a full human being.

Some empirical findings supported the role of dehumanization as one of the components of objectification. For example, observers were less likely to attribute human-related traits to objectified women (Vaes et al., 2011; Puvia and Vaes, 2013). Some researchers also showed that sexualization mainly contributed to animalistic dehumanization, whereas appearance-focused objectification mainly contributed to mechanistic dehumanization (Morris et al., 2018). As for those victims of objectification, employees who experienced higher levels of objectification at work reported that they experienced increased levels of organizational dehumanization (Sainz and Baldissarri, 2021). As such, objectified individuals would also experience dehumanization in their objectification experience.

It is reasonable to expect that the dehumanization component of objectification could also be internalized and result in self-dehumanization. Some findings support the link between dehumanization experience and self-dehumanization. For example, Song et al. (2022) revealed that conferring individuals with numerical identifications (i.e., a form of dehumanization), would trigger a higher level of self-dehumanization among them. Recently, other researchers detected that organizational dehumanization in a particular organization would motivate its staff to engage in more surface actions, and then make them carry a higher level of mechanistic self-dehumanization (Nguyen et al., 2022). With that being said, while being objectified, individuals would internalize the dehumanization component of their objectification experience, which causes them to regard themselves as less human.

Moreover, objectification could also contribute to self-dehumanization, when it is considered from another perspective. As it found by Renger et al. (2016), participants who received less intragroup respect rated themselves lower on human traits. Additionally, Yang et al. (2015) found that individuals in a powerless position rated themselves as less human. From a macroscopical viewpoint, those who experienced more maltreatment during their childhood would engage in greater self-dehumanization even after becoming adolescents (Jiang et al., 2021). Some other researchers even found that merely living in an environment with polluted air could make individuals engage in more self-dehumanization (Shi et al., 2022). Those findings all suggest that receiving maltreatment could make individuals perceive less humanness in themselves. Given the disadvantaged position that objectification puts its victims in (Zhang et al., 2023), objectification may also result in self-dehumanization if it is regarded as a kind of interpersonal maltreatment. More directly, despite a limited sample size (*n* = 62) and less reliable measurement (α < 0.70), it has been found that victims of workplace objectification rate themselves less warm, less competent, and less human, after the objectification experience (Loughnan et al., 2017; Study 2).

In sum, people who have experienced objectification are more likely to employ greater self-dehumanization. Notably, in the current research, we focus on self-dehumanization following objectification. Rather than self-instrumentality (i.e., individuals debase themselves to others’ tools willingly), because the former is more reasonably linked to prosociality. In the self-dehumanization process we theorized, objectified individuals would self-perceive as nonhuman beings without warmth, agency, rationality, and other features of humanness. Thus, we hypothesized that objectification would lead to self-dehumanization.

### The self-dehumanization and declined prosociality

In general, to maintain a positive self-image, individuals tend to attribute higher levels of humanness to themselves compared to others (Haslam et al., 2005). However, people would also employ self-dehumanization from time to time, in which they perceive themselves as less human. Employing self-dehumanization would lead to several negative impacts. For example, a recent study found that participants who reported greater self-dehumanization also reported higher levels of anxiety, depression, and less self-efficacy to refuse alcohol (Fontesse et al., 2021). Contrary to self-dehumanization, Ruttan and Lucas (2018) found that self-humanization was associated with reduced prioritization of money, supporting the idea that self-humanization has implications for behavior.

More importantly, self-dehumanization could also result in other interpersonal negative effects, especially making individuals less prosocial. As a core aspect of humanness, morality is associated strongly with the perception of humanization (e.g., Brandt and Reyna, 2011; Haslam et al., 2011). Self-dehumanization, as a result of objectification, may lead individuals to feel less obligated to adhere to moral standards associated with humanness, which can subsequently decrease their motivation to engage in prosociality. As found by Bastian et al. (2011), dehumanization could decrease individuals’ moral agency (which motivates individuals to conduct moral behaviors). Hence, for self-dehumanized individuals, their moral agency would decline, which restrains their prosociality. Empirically, Kouchaki et al. (2018) found that self-dehumanization could cause individuals to conduct increased immoral and antisocial behaviors. Therefore, we proposed that individuals’ self-dehumanization could decrease their prosociality.

Given the theoretical framework of self-dehumanization as a consequence of objectification experience, and the evidence suggesting its negative impact on prosociality, we hypothesized that the individuals’ self-dehumanization induced by their objectification experience could account for the negative relationship between objectification and prosociality.

### Current research

In the present research, we aimed to test the negative effect of nonsexual objectification on individuals’ prosociality, and to examine whether self-dehumanization could account for such an effect. We started testing our hypotheses with two correlational studies (Studies 1 and 2). We then manipulated participants’ objectification experience (Studies 3 and 4) involving both aspects of objectification (instrumentality and dehumanization), and examined the mediating role of self-dehumanization. Additionally, we also compared the mediating effects of self-dehumanization and relative deprivation in accounting for our hypothesized negative effect of objectification on prosociality (Studies 1, 2, and 4).

## Study 1

In this study, we tested the relation between objectification experience and prosociality using a correlational design, as well as the mediating role of self-dehumanization and relative deprivation. We predicted there would be a negative association between objectification and prosocial intention, as well as the mediating role of relative deprivation, and that the mediating effect of self-dehumanization can also be detected.

### Participants

In total, 250 American participants were recruited using Amazon’s Mechanical Turk (Rand, 2012), and they participated in an exchange for 0.3$ USD, while 21 were excluded for failing the attention check (i.e., “Please choose ‘*strongly disagree*’ for the current item”; same for the following studies). A sensitivity test (Faul et al., 2007) showed that our final sample size of 229 (137 women, *M*_age_ = 44.56, *SD*_age_ = 14.2) could provide 80% power to detect an effect of *R^2^* = 0.055 (small-medium; α = 0.05). Among them, 75.1% were White, 6.1% were African American, 5.2% were Asian, 6.6% were Latin, 5.2% were from other ethnicities, while another 1.7% preferred not to answer.

### Procedures and measurements

Generally, after consent forms, participants responded to several different scales. Firstly, participants’ objectification experience was tested. Then they rated themselves on relative deprivation and self-dehumanization measurements. Next, they reported their prosocial intention. In the end, participants’ demographic information was also collected (i.e., gender, race, age, and subjective socioeconomic status).

**Objectification experience** (*M* = 3.62, *SD* = 1.41, α = 0.60). On two items adapted from past research (Gruenfeld et al., 2008; i.e., “Other people’s relationship with me is important to them because it would help them accomplish their goals” and “If the condition changed and I would not be helpful anymore, my relationship with other people probably would not continue”), participants expressed the extent to which objectification they had experienced, and they responded on a scale ranging from *strongly disagree* (i.e., “1”) to *strongly agree* (i.e., “7”) for each item.

**Self-dehumanization** (*M* = 1.67, *SD* = 1.09, α = 0.79). On two items adapted from past research (Bastian and Haslam, 2010; i.e., “I feel like I am mechanical and cold, like a robot” and “I feel like I lack self-restraint. Like an animal”), participants indicated the extent to which they regard themselves as nonhuman, and they responded on a scale ranging from *strongly disagree* (i.e., “1”) to *strongly agree* (i.e., “7”) for each item.

**Relative deprivation** (*M* = 2.92, *SD* = 1.64, α = 0.85). We adapted two items from the Personal Relative Deprivation Scale developed by Callan et al. (2017; i.e., “I feel deprived when I think about what I have compared to what other people like me have” and “I feel resentful when I see how prosperous other people like me seem to be”). Participants reported their feelings of relative deprivation, and they responded on a scale ranging from *strongly disagree* (i.e., “1”) to *strongly agree* (i.e., “7”) for each item.

**Prosocial intention** (*M* = 1.67, *SD* = 1.09, α = 0.87). We adapted four items from the Prosocial Behavioral Intentions Scale (Baumsteiger and Siegel, 2018; e.g., “Help care for someone sick”). Participants expressed their willingness to do each prosocial behavior, and they responded on a scale ranging from *not at all* (i.e., “1”) to *very much so* (i.e., “7”) for each item.

### Results and discussion

After participants’ demographics were controlled, participants’ objectification experience negatively predicted their prosocial intention, *β* = −0.139, *t* = −2.26, *p* = 0.025, 95% CI [−0.259, −0.018], *R^2^* = 0.209. This means that when individuals experience more objectification, they would hold lower prosocial intention. Additionally, there were also significant associations between objectification and self-dehumanization, *β* = 0.325, *t* = 5.15, *p* < 0.001, 95% CI [0.201, 0.449], *R^2^* = 0.161, as well as objectification and relative deprivation, *β* = 0.283, *t* = 4.67, *p* < 0.001, 95% CI [0.164, 0.402], *R^2^* = 0.229. It indicated that more objectified individuals would employ more self-dehumanization and feel more relatively deprived.

Moreover, participants’ relative deprivation negatively predicted their prosocial intention, *β* = −0.194, *t* = −3.01, *p* = 0.003, 95% CI [−0.320, −0.0.067], *R^2^* = 0.223. Furthermore, participants’ self-dehumanization negatively predicted their prosocial intention, *β* = −0.301, *t* = −5.13, *p* < 0.001, 95% CI [−0.417, −0.186], *R^2^* = 0.276. These results revealed that individuals who dehumanized themselves more strongly and those who felt more relatively deprived would express lower prosocial intention (see Table 1 for the correlation matrix).

**Table 1:** Correlations matrix of the variables in Study 1\Study 2 (*N* = 229\233).

No.	Variables	1	2	3	4	5	6	7	8
1	Objectification Experience	——							
2	Self-dehumanization	0.361***\0.311***	——						
3	Relative Deprivation	0.337***\0.300***	0.382***\0.437***	——					
4	Prosocial Intention	−0.213*\−0.151*	−0.368***\−0.516***	−0.259***\−0.276***	——				
5	Gender	−0.102\−0.091	−0.054\−0.129	0.012\0.066	0.313***\0.213**	——			
6	Race	−0.013\−0.104	−0.037\−0.042	0.069\0.023	0.108\−0.070	0.100\−0.024	——		
7	Subjective SES	−0.028\−0.138*	0.098\0.065	−0.189**\−0.012	0.213**\−0.067	0.010\0.042	0.001\−0.143*	——	
8	Age	−0.201**\-0.162*	−0.222**\-0.245***	−0.353***\−0.172**	0.205*\−0.182**	−0.041\−0.009	−0.049\0.029	0.097\−0.069	——

*p < 0.05; **p < 0.01; ***p < 0.001.

**Figure 1:**
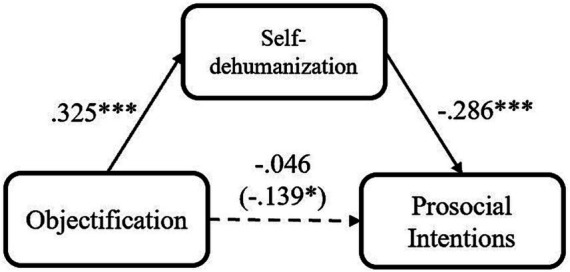
Mediation models of participants’ objectification experience on their prosocial intention via self-dehumanization, for Study 1 (*p < 0.05; **p < 0.01; ***p < 0.001).

Based on that, we explored the mediating role of participants’ self-dehumanization and relative deprivation. We conducted several mediation analyses using PROCESS Model 4 (Hayes, 2012; 5,000 iterations, bias-corrected), in which participants’ demographics were included as covariates. Firstly, as we predicted, self-dehumanization mediated the relationship between participants’ objectification experience and prosocial intention, indirect effect = −0.093, *SE* = 0.016, 95% CI [−0.098, −0.020] (Figure 1). Additionally, consistent with the previous finding (Zhang et al., 2023), relative deprivation could also account for the relationship between participants’ experience of objectification and their prosocial intention, indirect effect = −0.046, *SE* = 0.013, 95% CI [−0.058, −0.001]. Importantly, the effect of self-dehumanization was still significant after relative deprivation being included as another covariate, indirect effect = −0.066, *SE* = 0.014, 95% CI [−0.076, −0.012]. Meanwhile, the effect of self-dehumanization (indirect effect = −0.086, *SE* = 0.016, 95% CI [−0.082, −0.020]) was stronger than the effect of relative deprivation (indirect effect = −0.026, *SE* = 0.012, 95% CI [−0.039, 0.007]) when they were included in an integrate mediation model. The results indicated that self-dehumanization and relative deprivation could both interpret the association between objectification and prosocial intention. Nevertheless, it seemed that self-dehumanization was a stronger mediator in the relation between objectification and prosocial intention.

In the present study, we provide more evidence for the negative relationship between objectification and prosociality. In line with the research by Zhang et al., (2023), individuals who experienced more objectification would be less prosocial. Importantly, we found that self-dehumanization could serve as a mechanism in the relationship between objectification and prosociality, whose effect was independent of relative deprivation and stronger than it. Specifically, as we theorized, objectified individuals would see themselves as less human, and such self-dehumanization would prevent them from expressing prosociality. However, in this study, we did not apply the full measurements of all the variables, which might decrease their reliability and validity. Given that, in the next study, we introduced more complete scales for further investigation.

## Study 2

In this study, with more reliable and more valid measurements, we replicated the findings in Study 1 using another correlational design. Consistent with Study 1, we also predicted that participants’ objectification experience could negatively predict their prosocial intention, while self-dehumanization could still serve as an independent mediator.

### Participants

In total, 250 American participants were recruited using Amazon’s Mechanical Turk (Rand, 2012), and they participated in exchange for 0.3$ USD, while 17 were excluded for failing the attention check. A sensitivity test (Faul et al., 2007) showed that our final sample size of 233 (132 women, *M*_age_ = 40.9, *SD*_age_ = 11.0) could provide 80% power to detect an effect of R^2^ = 0.054 (small-medium; α = 0.05). Among them, 70.4% were White, 9.0% were African American, 5.6% were Asian, 3.0% were Latin, 10.7% were from other ethnicities, while another 1.3% preferred not to answer.

### Procedures and measurements

Generally, after completing the consent forms, participants encountered different scales separately. Firstly, participants’ objectification experience was assessed. Then, their self-dehumanization and relative deprivation were measured. Afterward, they reported their prosocial intention. Finally, participants’ demographic information (same as Study 1) was collected.

**Objectification experience** (*M* = 3.89, *SD* = 0.96, α = 0.81). On eight items adapted from past research (Gruenfeld et al., 2008; e.g., “Other people tend to contact me only when they need something from me”). Participants expressed the extent to which objectification they had experienced, and they responded on a scale ranging from *strongly disagree* (i.e., “1”) to *strongly agree* (i.e., “7”) for each item.

**Self-dehumanization** (*M* = 2.49, *SD* = 0.87, α = 0.84). On 10 items adapted from past research (Bastian and Haslam, 2010; e.g., “I feel that I am mechanical and cold, like a robot”). Participants indicated the extent to which they regard themselves as nonhuman, and they responded on a scale ranging from *strongly disagree* (i.e., “1”) to *strongly agree* (i.e., “7”) for each item.

**Relative deprivation** (*M* = 3.55, *SD* = 1.18, α = 0.76). We applied the five-item Personal Relative Deprivation Scale derived from Callan et al. (2017; e.g., “I feel deprived when I think about what I have compared to what other people like me have”). Participants reported their feelings of relative deprivation, and they responded on a scale ranging from *strongly disagree* (i.e., “1”) to *strongly agree* (i.e., “7”) for each item.

**Prosocial intention** (*M* = 8.24, *SD* = 1.67, α = 0.96). On a 14-items prosocial tendency scale developed by Jiang and Sedikides (2021; e.g., “I feel I would do what I can to help others avoid getting into trouble). Participants expressed their willingness to do each prosocial behavior, and they responded on a scale ranging from *not at all* (i.e., “1″) to *very much so* (i.e., “11″) for each item.

### Results and discussion

Overall, after participants’ demographics were controlled, participants’ objectification experience predicted their prosocial intention negatively, *β* = −0.130, *t* = −1.99, *p* = 0.047, 95% CI [−0.258, −0.001], *R^2^* = 0.106. In line with our prediction, the current results indicated that when individuals suffered from more objectification, they would express a lower prosocial intention. In addition, participants’ objectification experience positively predicted their self-dehumanization, *β* = 0.284, *t* = 4.48, *p* < 0.001, 95% CI [0.159, 0.408], *R^2^* = 0.156, and relative deprivation, *β* = 0.298, *t* = 4.61, *p* < 0.001, 95% CI [0.171, 0.426], *R^2^* = 0.118. It means that more objectified individuals were more likely to regard themselves as nonhuman and felt more relatively deprived.

Moreover, participants’ self-dehumanization and relative deprivation negatively related to their prosocial intention. Specifically, there was a negative association between participants’ self-dehumanization and prosocial intention, *β* = −0.482, *t* = −8.33, *p* < 0.001, 95% CI [−0.596, −0.368], *R^2^* = 0.303, as well as a negative relation between participants’ relative deprivation and prosocial intention, *β* = −0.267, *t* = −4.31, *p* = 0.001, 95% CI [−0.390, −0.145], *R^2^* = 0.159. It was revealed that individuals with a higher magnitude of self-dehumanization and relative deprivation tend to express lower prosocial intention (see Table 1 for the correlation matrix).

**Figure 2:**
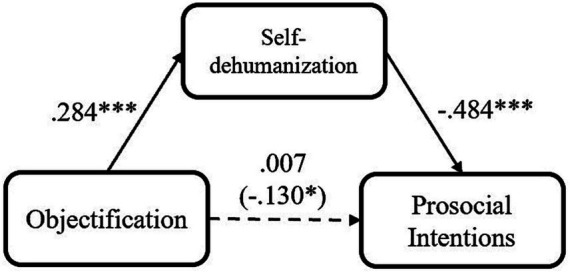
Mediation models of participants’ objectification experience on their prosocial intention via self-dehumanization, for Study 2 (*p < 0.05; **p < 0.01; ***p < 0.001).

Based on that, the we consistently explored the mediating roles of self-dehumanization and relative deprivation. We conducted several mediation analyses using PROCESS Model 4 (Hayes, 2012; 5,000 iterations, bias-corrected), after participants’ demographics were included as covariates. Firstly, self-dehumanization also mediated the relationship between participants’ objectification experience and prosocial intention, indirect effect = −0.137, *SE* = 0.060, 95% CI [−0.356, −0.121] (Figure 2). Additionally, relative deprivation mediated the relation between participants’ objectification experience and prosocial intention, indirect effect = −0.075, *SE* = 0.043, 95% CI [−0.215, −0.046]. Further, the effect of self-dehumanization was still significant even after relative deprivation being included as another covariate, indirect effect = −0.078, *SE* = 0.051, 95% CI [−0.236, −0.035]. Meanwhile, the effect of self-dehumanization (indirect effect = −0.128, *SE* = 0.058, 95% CI [−0.336, −0.108]) was stronger than the effect of relative deprivation (indirect effect = −0.025, *SE* = 0.034, 95% CI [−0.110, 0.022]) when they were included in an integrate mediation model. These results indicated that self-dehumanization (vs. relative deprivation) is a stronger mediator in the relation between objectification and prosocial intention, and its effect is independent of the effect of relative deprivation.

In this study, with more intact measurements, we replicated the negative association between objectification and prosocial intention, as well as the mediating effect of relative deprivation and self-dehumanization. Moreover, the mediating effect of self-dehumanization was still found to be stronger than relative deprivation, and independent of relative deprivation. Nevertheless, in both Studies 1 and 2, all the findings were based on correlational data. To provide causal evidence, we will manipulate objectification in the next study.

## Study 3

For this study, we manipulated objectification with a recall paradigm, in which both aspects of objectification (i.e., instrumentality and dehumanization) were involved. Additionally, as objectification is a negative experience, it may also induce negative emotion among objectification targets (Poon et al., 2020), which might in turn decrease their prosociality. Therefore, the potential influence of negative emotion should be controlled, and we also measured this variable and included it as a covariate in our data analyses (same for Study 4). We predicted that the manipulated objectification experience could induce decreased prosociality among participants, and the mediating role of self-dehumanization could still be detected. We also predicted that these aforementioned effects can still be detected even after including negative emotion as another covariate.

### Participants

In total, 250 American participants were recruited using Amazon’s Mechanical Turk (Rand, 2012), and they participated in exchange for 0.3$ USD, while 20 were excluded for failing the attention check. A sensitivity test (Faul et al., 2007) showed that our final sample size of 230 (164 women, *M*_age_ = 40.5, *SD*_age_ = 12.3) could provide 80% power to detect an effect of *η*_p_^2^ = 0.033 (small-medium; α = 0.05). Among them, 63.9% were White, 7.8% were African American, 7.0% were Asian, 5.7% were Latin, 12.6% were from other ethnicities, while another 3.0% preferred not to answer.

### Procedures and measurements

In the beginning, participants were informed that they would take part in a recall task to test their memory. After consent forms, participants were randomly assigned to two experimental conditions. In the objectification condition, participants were asked to recall an objectification experience (i.e., an experience they were treated as a tool or object to achieve others’ goals and benefits while their feelings and needs were ignored). In contrast, their counterparts in the non-objectification condition were asked to recall an experience at a grocery store. Next, they were presented with three manipulation check items (e.g., “In the above task, people treat me as an instrument to complete their own jobs”; *M* = 3.78, *SD* = 2.45, α = 0.98).

Then, all participants were asked to report their self-dehumanization (*M* = 3.09, *SD* = 1.06, α = 0.81) on 10 items adapted from previous research, which was identical to Study 2. After that, participants’ negative emotions (*M* = 3.42, *SD* = 1.77, α = 0.92) were measured (e.g., “I feel sad”; 1 = *strongly disagree*, 7 = *strongly agree*). Next, they were confronted with a scale to test their prosocial intention (*M* = 8.43, *SD* = 1.78, α = 0.96), which was identical to Study 2. Finally, participants reported their demographic information (same as Study 1), and then they were thanked and debriefed.

### Results and discussion

According to the manipulation check, participants in the objectification condition (*M* = 6.08, *SD* = 1.16) reported that they experienced more objectification compared to their counterparts in the neutral condition (*M* = 2.14, *SD* = 1.69), *t* (228) = −19.72, *p* < 0.001, *d* = −2.64, 95% CI [−4.33, −3.54]. The results indicated that our manipulation was effective.

However, after participants’ negative emotions and their demographics were included as covariates, an ANCOVA analysis revealed no significant discrepancy between participants in the objectification condition (*M* = 8.54, *SD* = 1.76) and the control condition (*M* = 8.36, *SD* = 1.80) on their prosocial intention, *F* (1, 224) = 1.49, *p* = 0.224, *η*_p_^2^ = 0.007. Nevertheless, another similar ANCOVA analysis indicated that participants in the objectification condition (*M* = 3.50, *SD* = 1.16) employed more self-dehumanization than their counterparts (*M* = 2.79, *SD* = 0.87), *F* (1, 224) = 7.45, *p* = 0.007, *η*_p_^2^ = 0.032. In addition, participants’ self-dehumanization negatively predicted their prosocial intention, *β* = −0.326, *t* = −2.61, *p* = 0.010, 95% CI [−0.572, −0.079], *R^2^* = 0.097.

**Figure 3:**
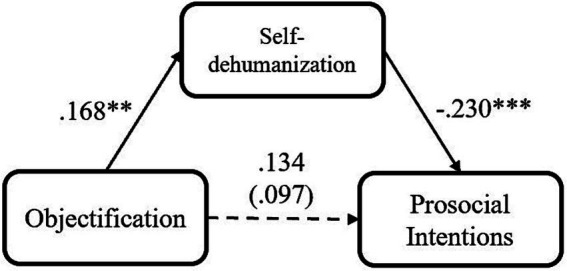
Mediation models of participants’ objectification experience on their prosocial intention via self-dehumanization, for Study 3 (objectification condition = 1, control condition = 0, *p < 0.05; **p < 0.01; ***p < 0.001).

Regarding that, we still tested the mediating role of self-dehumanization. After participants’ negative emotions and their demographics were included as covariates, a mediation analysis was conducted with PROCESS Model 4 (Hayes, 2012; 5,000 iterations, bias-corrected). Consistent with the results of Studies 1 and 2, participants’ self-dehumanization consistently mediated the relationship between their objectification experience and their prosocial intention, indirect effect = −0.037, *SE* = 0.066, 95% CI [−0.262, −0.004] (Figure 3). It means that objectification experience induces more self-dehumanization among its victims, while such self-dehumanization could then lead to their lower prosocial intention. In contrast, participants’ negative emotions could not play a similar mediating role as self-dehumanization, indirect effect = −0.032, *SE* = 0.101, 95% CI [−0.314, 0.082]. As a result, the results indicate that the mediating effect of self-dehumanization is independent from both relative deprivation and negative emotion, and negative emotion cannot interpret the relationship between objectification and declined prosociality.

Different from our prediction, we did not detect the main effect of objectification on prosocial intention. Besides, the effect of objectification on self-dehumanization was underpowered. Nevertheless, we still found that objectification could influence prosocial intention indirectly via self-dehumanization, while the mediation model (objectification → self-dehumanization → decreased prosocial intention) could provide 96% power for the indirect effect we detected (based on Monte Carlo Power Analysis, Schoemann et al., 2017). These insignificant or underpowered effects may result from the recall paradigm, as there was a large variance in participants’ recall. Thus, in the next study, we employed an imagining task adapted from Dai et al. (2023), in which the magnitude of objectification was relatively fixed and the two aspects of objectification were equally emphasized. Additionally, similar to Studies 1 and 2, we measured participants’ relative deprivation to further confirm the distinct mediating effect of self-dehumanization.

## Study 4

In this study, we introduced another paradigm to manipulate objectification and retest its effect on participants’ prosociality. Besides, we measured participants’ negative emotion and included it as a covariate in data analyses. As before, we predicted that the relation between objectification and prosociality could be replicated, and the distinct mediating role of self-dehumanization could also be detected. We also predicted that these aforementioned effects can still be detected even after including negative emotion as another covariate.

### Participants

In total, 200 Chinese participants were recruited using a Chinese online platform called Credaom, and they participated in exchange for ¥3 RMB (around $0.4 USD), while 15 were excluded for failing the attention check. A sensitivity test (Faul et al., 2007) showed that our final sample size of 185 (86 women, *M*_age_ = 22.3, *SD*_age_ = 3.88) could provide 80% power to detect an effect of *η*_p_^2^ = 0.041 (small-medium; α = 0.05) for ANCOVA tests.

### Procedures and measurements

In the beginning, participants were informed that they would take part in an imagining task to test their imagination. After consent forms, participants were randomly assigned to two experimental conditions. In the high objectification condition, participants were asked to imagine a serious objectification experience during their work (i.e., they are treated as instruments by their colleagues and superiors, while their needs and feelings were ignored; for details, please see Supplementary Material). Differently, their counterparts in the low objectification condition were asked to imagine a similar experience in which they were treated relatively decently, and their feelings were cared for. Next, they were encountered with three manipulation check items (e.g., “In the above task, people treat me as an instrument to complete their own jobs”; *M* = 4.72, *SD* = 2.01, α = 0.94).

Then, all participants reported their self-dehumanization (*M* = 4.01, *SD* = 1.44, α = 0.91), and the measurement was identical to Study 2, but in Chinese (Bastian and Haslam, 2010; e.g., “I feel that I am superficial like I have no depth”; 1 = *strongly disagree*, 7 = *strongly agree*). After that, their relative deprivation (*M* = 4.19, *SD* = 1.75, α = 0.79; i.e., “I feel deprived when I think about what I have compared to what other people like me have” and “I feel resentful when I see how prosperous other people like me seem to be”; 1 = *strongly disagree*, 7 = *strongly agree*) and negative emotions (*M* = 4.19, *SD* = 2.02, α = 0.93; i.e., “I feel bad” and “I feel sad”; 1 = *strongly disagree*, 7 = *strongly agree*) were measured. Next, they were encountered with a scale to measure their prosocial intention (*M* = 5.01, *SD* = 1.34, α = 0.96), which was identical to Study 2. Finally, participants’ demographic information was collected (i.e., gender, age, and subjective SES), and then they were thanked and debriefed.

### Results and discussion

According to the manipulation check, participants in the high objectification condition (*M* = 6.34, *SD* = 0.87) reported that they experienced more objectification compared to their counterparts in the low objectification condition (*M* = 3.09, *SD* = 1.43), *t* (183) = −18.75, *p* < 0.001, *d* = −2.76, 95% CI [−3.25, −2.26]. The results indicated that our manipulation was effective.

Based on that, after including participants’ negative emotions and their demographics as covariates, we conducted several ANCOVA analyses to test the effect of objectification on participants’ self-dehumanization, relative deprivation, and prosocial intention. As predicted, compared to participants in the low objectification condition (*M* = 5.42, *SD* = 0.89), participants in the high objectification condition (*M* = 4.61, *SD* = 1.58) expressed significantly lower prosocial intention, *F* (1, 179) = 17.21, *p* < 0.001, *η*_p_^2^ = 0.088. The results also indicated that participants in the high objectification condition (*M* = 4.98, *SD* = 1.19) tended to employ greater self-dehumanization than their counterparts (*M* = 3.02, *SD* = 0.92), *F* (1, 179) = 158.08, *p* < 0.001, *η*_p_^2^ = 0.469. Meanwhile, those in the high objectification condition (*M* = 5.13, *SD* = 1.40) also reported higher levels of relative deprivation than their counterparts (*M* = 3.24, *SD* = 1.55), *F* (1, 179) = 72.63, *p* < 0.001, *η*_p_^2^ = 0.289.

**Figure 4:**
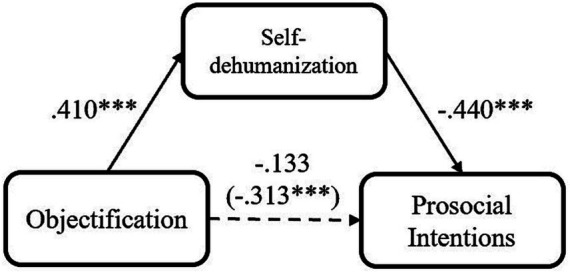
Mediation models of participants’ objectification experience on their prosocial intention via self-dehumanization, for Study 4 (high objectification condition = 1, low objectification condition = 0, *p < 0.05; **p < 0.01; ***p < 0.001).

To test the mediation role of self-dehumanization and relative privation, after participants’ negative emotions and their demographics were included as covariates, we conducted several mediation analyses with PROCESS Model 4 (Hayes, 2012; 5,000 iterations, bias-corrected). Consistent with the results of previous studies, the mediating effects of self-dehumanization in the relation between objectification and prosocial intention were significant, indirect effect = −0.180, *SE* = 0.135, 95% CI [−0.747, −0.219] (Figure 4). It means that objectification experience could make individuals employ more self-dehumanization, and such self-dehumanization could in turn decrease their prosocial intention. Additionally, relative deprivation mediated the relation between participants’ objectification experience and their prosocial intention, indirect effect = −0.062, *SE* = 0.081, 95% CI [−0.324, −0.007]. Even after relative deprivation was controlled, the effect of self-dehumanization was still significant, indirect effect = −0.143, *SE* = 0.121, 95% CI [−0.619, −0.137]. Meanwhile, the effect of self-dehumanization (indirect effect = −0.165, SE = 0.133, 95% CI [−0.703, −0.151]) was stronger than the effect of relative deprivation (indirect effect = −0.040, SE = 0.073, 95% CI [−0.248, 0.036]) when they were included in an integrate mediation model. It indicated that the effect of self-dehumanization was stronger than relative deprivation, and independent from it. Moreover, participants’ negative emotions could not account for the relation between objectification and prosociality, indirect effect = 0.013, *SE* = 0.163, 95% CI [−0.282, 0.355]. It means that the mediating effect of self-dehumanization is independent from both relative deprivation and negative emotion, and negative emotion cannot interpret the relationship between objectification and declined prosociality.

With a new paradigm and another group of participants with different cultural backgrounds, we detected the effects of objectification on prosocial intention. We also found that self-dehumanization could account for the negative effect of individuals’ objectification on their prosocial intention. Additionally, in line with Studies 1 and 2, we also showed that, in the relationship between objectification and prosocial intention, self-dehumanization was a stronger and distinct mediator compared to relative deprivation. Moreover, consistent with Study 3, these aforementioned effects were independent from individuals’ negative emotion induced by objectification experience.

## General discussion

We conducted four studies were conducted to depict a clearer view of the relationship between objectification and prosociality. In Study 1, participants who reported more objectification experience would express lower prosocial intention, whereas self-dehumanization could account for this association. Study 2 replicated the findings in Study 1 with more improved measurements. Further, in Study 3, by manipulating objectification with a recall paradigm that involves both aspects of objectification (instrumentality and dehumanization), we repeatedly detected the mediating role of self-dehumanization in the relationship between objectification and prosocial intention, although the main effect of objectification on prosocial intention was not significant. Lastly, in Study 4, using a newly developed imagining task (Dai et al., 2023) and recruiting participants from another cultural background, we detected the negative impact of objectification on prosocial intention as well as the mediating role of self-dehumanization. Additionally, Studies 1, 2, and 4 converged in showing that self-dehumanization is a stronger mediator than relative deprivation, a previously reported mediator. Moreover, in studies 3 and 4, we controlled for the potential contribution of negative emotion, thus our reported effects were independent from individuals’ emotional variation during objectification experience. These findings, along with recent literature, delineate the relationship between objectification and prosociality as well as its underlying mechanisms.

### Implications of the present research

Our current research could make several contributions to the literature. Firstly, our findings strongly support the internalization process associated with people’s objectification experience using diverse research designs and participants from different cultural backgrounds, which is in line with the argument that dehumanization is a critical component of objectification (e.g., Nussbaum, 1995; Baldissarri et al., 2022).

Secondly, consistent with a list of previous researchers (e.g., Baldissarri et al., 2021), our findings support these arguments that objectification is a negative experience that could lead to negative impacts on both intrapersonal and interpersonal levels. In particular, consistent with former findings (Zhang et al., 2023), we replicated the negative link between greater objectification and declined prosociality. Nevertheless, the effect of objectification on prosociality could be relatively weaker occasionally. This suggests that minor objectification could not obviously result in diminished prosocial expressions, which might make people neglect its negative impacts from time to time. However, regarding those indirect mediation models, we found that even nonserious objectification can make individuals employ greater self-dehumanization, and such self-dehumanization could then decrease their prosociality indirectly.

Moreover, consistent with what was revealed by Kouchaki et al. (2018), our research further supports the relationship between self-dehumanization and unethical expressions. Our research primarily focuses on the impact of self-dehumanization on restraining individuals’ positive actions. Together with their findings, our research suggests a potential model to understand the harmful effects of self-dehumanization on individuals’ interpersonal relations. Specifically, it means that self-dehumanization can impair one’s interpersonal relationships through both active (i.e., increase antisociality) and passive (i.e., decrease prosociality) paths. However, some scholars received contrasting findings, and they found a positive relationship between individuals’ self-dehumanization and their volunteering (Bastian et al., 2013). In line with the arguments from Kouchaki et al. (2018), we also hold the opinion that the different consequences of self-dehumanization were determined by its antecedents. Specifically, if self-dehumanization arises from immoral behaviors that individuals have conducted toward others, it will elicit some virtuous behaviors to restore their humanness. Conversely, if self-dehumanization results from maltreatment enforced by others (e.g., objectification or ostracism), it will make those victims more antisocial and less prosocial.

Furthermore, as we consistently found in Studies 1, 2, and 4, the mediating effect of self-dehumanization was stronger than and distinct from relative deprivation. Relative deprivation is a more common experience among individuals who have been mistreated, and it could account for various negative behaviors after experiencing maltreatment. For instance, previous research has shown that ostracism can trigger feelings of relative deprivation, whereas such relative deprivation could motivate them to be more aggressive (Jiang and Chen, 2020). Additionally, on the intergroup level, Muslims born in foreign countries reported experiencing higher levels of group-based relative deprivation, which in turn could lead to increased intentions for violent behaviors (Obaidi et al., 2019). In contrast, self-dehumanization was usually observed to be induced by experiences including being dehumanized, suggesting that it may be more susceptible to the effects of objectification. Therefore, self-dehumanization could serve as a better internal process to help us understand the relationship between objectification and prosociality.

### Limitations and future directions

Despite these abovementioned potential contributions, our research is not without limitations, which may suggest venues for future investigations. First of all, although we found a consistent effect of objectification on self-dehumanization, as well as the following declined prosociality, we have not explored other potential negative impacts that result from objectification-induced self-dehumanization. For instance, as self-dehumanized individuals are less reluctant to immoderate drinking (Fontesse et al., 2021), it is possible that objectified individuals are more likely to suffer from problematic drinking behavior. It suggests that workplace objectification, rather than just work pressure, may be a factor in the development of alcohol dependence among individuals in the workplace. Bastian et al. (2013) found that individuals may engage in behaviors to restore their sense of humanness when experiencing self-dehumanization. Given that objectification can lead to self-dehumanization, some objectified individuals may engage in people-pleasing behaviors as a way to restore their sense of humanness, which may cast other potential negative consequences, such as eating disorders (Exline et al., 2012). Additionally, self-dehumanization could reduce individuals’ self-efficacy (Fontesse et al., 2021). Given the strong linkage between self-efficacy and work-related performance (e.g., Stajkovic and Luthans, 1998), self-dehumanization may serve as the mechanism for the negative effect of objectification on task performance, as reported by Baldissarri and Andrighetto (2021). As such, future research will provide a better understanding of the above topics.

Additionally, we did not explore the potential strategies to buffer the negative effect of objectification on prosociality. As reciprocity plays an important role to maintain a cooperative society and reinforce altruism (e.g., Fehr and Fischbacher, 2003), objectification accompanied by positive reciprocity may have fewer negative effects. For example, despite the negative impacts of workplace objectification (e.g., Dai et al., 2023), objectified individuals may still get some benefits or rewards, which allows objectification, especially workplace objectification, to be paid off more or less. As a result, even just the working environment could motivate individuals to objectify others (Belmi and Schroeder, 2021), and daily objectification may have less noticeable negative consequences, if it comes with positive reciprocity.

Moreover, current findings are not enough to make a causal conclusion regarding the mediation model we detected. Previous research has shown that conducting ostracism on others can lead to self-dehumanization (Bastian et al., 2013), which suggests that behaving immorally could result in self-dehumanization. Hence, there is an alternative that self-dehumanization might be the consequence of reduced prosociality, as individuals may see themselves as less human because of their won declined prosociality after objectification. However, most of our data seems not supportive enough for such an alternative. Specifically, the mediating effects of prosocial intention in the relation between objectification and self-dehumanization were not significant in Study 1, indirect effect = 0.033, *SE* = 0.020, 95% CI [−0.001, 0.080], in Study 2, indirect effect = 0.054, *SE* = 0.027, 95% CI [−.107e-5, 0.108], and in Study 3, indirect effect = −0.035, *SE* = 0.028, 95% CI [−0.089, 0.019]. Even in Study 4, the indirect of prosocial intention, indirect effect = 0.189, *SE* = 0.071, 95% CI [0.050, 0.328], was much weaker than self-dehumanization (indirect effect = −0.483). It means the model we proposed is much better than the alternative one. Thus, we still concluded that self-dehumanization should occur before individuals’ decreased prosociality in objectification experiences. To provide more direct causal evidence for the mediating role of self-dehumanization, future research may consider manipulating objectification and self-dehumanization. It allows for a more convincing causal test of our proposed mediational process than our current approach.

Furthermore, although self-dehumanization is apparently a negative result of objectification, its effect on further negative consequences (e.g., declined prosociality) could be qualified by contexts. For instance, Schumann and Walton (2022) found that forgiving perpetrators could help victims of negative experiences promote their humanness. Further, Vaes and Bastian (2021) revealed that the above effect also occurred among self-dehumanized victims. Additionally, self-dehumanized individuals could still increase their humanness through the above approach even without perpetrators’ apology (Vaes et al., 2022). Therefore, the prosociality of self-dehumanized objectification-victims may not decrease if they are offered a chance to forgive their perpetrators who objectified them. In this way, the negative effect of objectification on prosociality could be limited. Future research could explore whether forgiveness could help self-dehumanized victims of objectification restore their humanness and then result in less drop in their prosociality.

Previous research has indicated the cultural differences in the domain of objectification (e.g., Wang et al., 2022). The effect of objectification on prosociality may also be qualified by cultural factors. For example, people with interdependent self-construal focus more on their interpersonal relationships than their counterparts (Markus and Kitayama, 1991), which might make them more vulnerable to objectification. Therefore, individuals with interdependent self-construal may employ greater self-dehumanization after experiencing objectification, which could in turn decrease their prosociality. Future researchers can explore whether self-construal and other cultural factors (e.g., individualism–collectivism, holistic-analytic cognition, etc.) could moderate the effects of objectification, and they may also recruit participants with different cultural backgrounds in one study for a cross-cultural comparison.

Finally, there were methodological limitations. For example, as we only focused on participants’ prosociality on an attitudinal level, the potential effect of objectification on individuals’ prosocial behaviors should be further investigated. Future research may utilize suitable paradigms, such as asking them to do repetitive tasks for charitable goals (Poulin et al., 2021), to assess the behavioral component of prosociality. Additionally, prosociality in people’s daily lives could also be observed through other attitudes and behaviors apart from helping, such as promoting social justice (e.g., Dik et al., 2012), serving in local communities (e.g., Christoph et al., 2014), or expressing compassion to others (e.g., Stevens and Taber, 2021). Future research may consider introducing other diversiform attitudes or behaviors to index prosociality, or even develop new measurements with high ecological validity to capture prosociality.

## Conclusion

Our four studies consistently supported the negative effect of objectification on prosociality. Importantly, we found that self-dehumanization could account for such an effect, whose mediating effect was stronger and distinct when compared with relative deprivation. Therefore, these findings contribute to a more comprehensive understanding of objectification by highlighting its dehumanizing aspect and to the literature on self-dehumanization by identifying another negative consequence.

## Data availability statement

The datasets presented in this study can be found in online repositories. The names of the repository/repositories and accession number(s) can be found at: https://osf.io/qwjzr/.

## Ethics statement

The studies involving humans were approved by Human Research Ethics Committee (HREC; EA1803011), The University of Hong Kong. The studies were conducted in accordance with the local legislation and institutional requirements. The participants provided their written informed consent to participate in this study.

## Author contributions

ZZ: Conceptualization, Data curation, Formal analysis, Investigation, Methodology, Project administration, Software, Validation, Writing – original draft, Writing – review & editing. ZC: Conceptualization, Funding acquisition, Investigation, Project administration, Resources, Supervision, Validation, Writing – review & editing, Methodology.
