# Supplementary material for: Nonhuman treatment reduces helping others: self-dehumanization as a mechanism
Source: Front Psychol. 2024 Mar 5;15:1352991. doi: 10.3389/fpsyg.2024.1352991 (PMC10948621; doi:10.3389/fpsyg.2024.1352991)
Supplement: Supplementary file 1 [file Data_Sheet_1.docx]

Supplementary Materials

Study Materials

[Study 1 2](#_Toc151906307)

[Study 2 4](#_Toc151906308)

[Study 3 8](#_Toc151906309)

[Study 4 11](#_Toc151906310)

# Study 1

**Measurement of Objectification Experience**

1. Other people’s relationship with me is important to them because it would help them accomplish their goals.

2. If the condition changed and I am helpful anymore, my relationship with other people probably cannot continue

(1=completely disagree, 7=completely agree)

**Measurement of Self-dehumanization**

1. I feel that I am mechanical and cold, like a robot.

2. I feel that I lack self-restraint, like an animal.

(1 = completely disagree, 7 = completely agree)

**Measurement of Relative Deprivation**

1. I feel deprived when I think about what I have compared to what other people like me have.

2. I feel resentful when I see how prosperous other people like me seem to be.

(1=completely disagree, 7=completely agree)

**Measurement of Prosocial Intention**

Please indicate your willingness to do the following behaviors.

1. Comfort someone after he\she experience a hardship.

2. Help someone find something lost, like their key or a pet.

3. Help care for someone sick.

4. Assist someone with a small task (e.g., help carry groceries, watch his\her things while he\she use the restroom).

(1 = not at all, 7 = very much so)

# Study 2

**Measurement of Objectification Experience**

1. Other people think more about what I can do for them than what they can do for me.

2. Other people tend to contact me only when they need something from me.

3. Other people interested in my feelings because they want to be close with me.*

4. Other people try to motivate me to do things that will help them succeed.

5. The relationship with me is important to other people because it helps them accomplish their goals.

6. Other people’s relationship with me is based on how much they enjoy our relationship, rather than how productive our relationship is.*

7. If the condition changed and I am helpful anymore, my relationship with other people probably cannot continue.

8. Other people really like me a lot even though I am not all that useful to them.*

(1=completely disagree, 7=completely agree, * items are reverse score items)

**Measurement of Self-dehumanization**

1. I feel that I am emotional, like I am responsive and warm.*

2. I feel like I am open-minded, like I can think clearly about things.*

3. I feel that I am superficial like I have no depth.

4. I feel that I am mechanical and cold, like a robot.

5. I feel that I am refined and cultured.*

6. I feel like I am connected to other people.*

7. I feel that I am rational and logical.*

8. I feel like I am incompetent.

9. I feel that I lack self-restraint, like animals.

10. I feel that I am intelligent.*

(1 = completely disagree, 7 = completely agree, * items are reverse score items)

**Measurement of Relative Deprivation**

1. I feel deprived when I think about what I have compared to what other people like me have.

2. I feel privileged compared to other people like me.*

3. I feel resentful when I see how prosperous other people like me seem to be.

4. When I compare what I have with what others like me have, I realize that I am quite well off.*

5. I feel dissatisfied with what I have compared to what other people like me have.

(1=completely disagree, 7=completely agree, * items are reverse score items)

**Measurement of Prosocial Intention**

1. I feel I would be responsive to those who are in need.

2. I feel I would try to help others.

3. I feel I would be pleased to help my friends/colleagues in their activities.

4. I feel I would like to share the things that I have with my friends.

5. I feel I would be available for voluntary activities to help those who are in need.

6. I feel I would help immediately those who are in need.

7. I feel I would do what I can to help others avoid getting into trouble.

8. I feel I would be willing to make my knowledge and abilities available to others.

9. I feel I would try to console those who are sad.

10. I feel I would easily lend money or other things to those in need.

11. I feel I would easily put myself in the shoes of those who are in discomfort.

12. I feel I would try to be close to and take care of those who are in need.

13. I feel I would easily share with friends any good opportunity that comes to me.

14. I feel I would spend time with those friends who feel lonely.

(1 = not at all, 11= extremely)

# Study 3

**Manipulation of Objectification Experience**

*For participants in objectification condition*:

In this section, please recall your WORST time in your life where you were OBJECTIFIED [i.e. you were treated as a tool or object to achieve other's goals and benefits while your feelings and needs were totally ignored].

*For participants in control condition*:

In this section, please recall an experience in which you visited a grocery store recently.

**Measurement of Self-dehumanization**

1. I feel that I am emotional, like I am responsive and warm.*

2. I feel that I am mechanical and cold, like a robot.

3. I feel that I am superficial like I have no depth.

4. I feel like I am open-minded, like I can think clearly about things.*

5. I feel like I am connected to other people.*

6. I feel that I am refined and cultured.*

7. I feel that I lack self-restraint, like animals.

8. I feel that I am rational and logical.*

9. I feel like I am incompetent.

10. I feel that I am intelligent.*

(1 = completely disagree, 7 = completely agree, * items are reverse score items)
**Measurement of Negative Emotion**

1. I feel happy.*

2. I feel bad.

3. I feel good.*

4. I feel sad.

(1 = completely disagree, 7 = completely agree, * items are reverse score items)

**Measurement of Prosocial Intention**

1. I feel I would be responsive to those who are in need.

2. I feel I would try to help others.

3. I feel I would be pleased to help my friends/colleagues in their activities.

4. I feel I would like to share the things that I have with my friends.

5. I feel I would be available for voluntary activities to help those who are in need.

6. I feel I would help immediately those who are in need.

7. I feel I would do what I can to help others avoid getting into trouble.

8. I feel I would be willing to make my knowledge and abilities available to others.

9. I feel I would try to console those who are sad.

10. I feel I would easily lend money or other things to those in need.

11. I feel I would easily put myself in the shoes of those who are in discomfort.

12. I feel I would try to be close to and take care of those who are in need.

13. I feel I would easily share with friends any good opportunity that comes to me.

14. I feel I would spend time with those friends who feel lonely.

(1 = not at all, 11= extremely)

# Study 4

**Manipulation of Objectification Experience**

*For participants in high-objectification condition*:

Imagine that you are a third-year undergraduate interning at a company.

You have an important course this semester, and you are looking forward to getting a good grade, so you study very hard. Recently, the course has a group assignment to complete. The quality of the group work greatly affects the course grade. At this point, several students invited you to form a group. However, after a period of working together, you find that your group members treat you as a tool to complete group assignments and get good grades.

In addition, in the company where you intern, you feel that your leader treats you as an object to reduce workload by giving you large amounts of work without any advice.

*For participants in low-objectification condition*:

Imagine that you are a third-year undergraduate interning at a company.

You have an important course this semester, and you are looking forward to getting a good grade, so you study very hard. Recently, the course has a group assignment to complete. The quality of the group work greatly affects the course grade. At this point, several students invited you to form a group. When working in the group, the group members listen carefully to the opinions and ideas of others. You find it very enjoyable to work together.

In the company where you intern, your leader not only provides some useful advice at work but also listens to your thoughts and wishes.

**Measurement of Self-dehumanization**

1. I feel that I am emotional, like I am responsive and warm.*

2. I feel that I am mechanical and cold, like a robot.

3. I feel that I am superficial like I have no depth.

4. I feel like I am open-minded, like I can think clearly about things.*

5. I feel like I am connected to other people.*

6. I feel that I am refined and cultured.*

7. I feel that I lack self-restraint, like animals.

8. I feel that I am rational and logical.*

9. I feel like I am incompetent.

10. I feel that I am intelligent.*

(1 = completely disagree, 7 = completely agree, * items are reverse score items)

**Measurement of Relative Deprivation**

1. I feel deprived when I think about what I have compared to what other people like me have.

3. I feel resentful when I see how prosperous other people like me seem to be.

(1=completely disagree, 7=completely agree)

**Measurement of Negative Emotion**

1. I feel bad.

2. I feel sad.

(1 = completely disagree, 7 = completely agree)

**Measurement of Prosocial Intention**

1. I feel I would be responsive to those who are in need.

2. I feel I would try to help others.

3. I feel I would be pleased to help my friends/colleagues in their activities.

4. I feel I would like to share the things that I have with my friends.

5. I feel I would be available for voluntary activities to help those who are in need.

6. I feel I would help immediately those who are in need.

7. I feel I would do what I can to help others avoid getting into trouble.

8. I feel I would be willing to make my knowledge and abilities available to others.

9. I feel I would try to console those who are sad.

10. I feel I would easily lend money or other things to those in need.

11. I feel I would easily put myself in the shoes of those who are in discomfort.

12. I feel I would try to be close to and take care of those who are in need.

13. I feel I would easily share with friends any good opportunity that comes to me.

14. I feel I would spend time with those friends who feel lonely.

(1 = not at all, 11= extremely)
